# A reasonable identification of the early recurrence time based on microvascular invasion for hepatocellular carcinoma after R0 resection: A multicenter retrospective study

**DOI:** 10.1002/cam4.5758

**Published:** 2023-03-06

**Authors:** Zong‐Han Liu, Zong‐Tao Chai, Jin‐Kai Feng, Yu‐Chao Hou, Xiu‐Ping Zhang, Zhen‐Hua Chen, Yan‐Jun Xiang, Wei‐Xing Guo, Jie Shi, Shu‐Qun Cheng

**Affiliations:** ^1^ Department of Hepatic Surgery VI, Eastern Hepatobiliary Surgery Hospital Second Military Medical University Shanghai China; ^2^ Cancer Center, Yueyang Hospital of Integrated Traditional Chinese and Western Medicine Shanghai University of Traditional Chinese Medicine Shanghai China; ^3^ Faculty of Hepato‐Biliary‐Pancreatic Surgery, Chinese People's Liberation Army (PLA) General Hospital Institute of Hepatobiliary Surgery of Chinese PLA Beijing China; ^4^ Department of General Surgery, Zhejiang Provincial Armed Police Corps Hospital Hangzhou China; ^5^ Department of Hepatobiliary Surgery, The First Affiliated Hospital Wenzhou Medical University Wenzhou China

**Keywords:** early recurrence, hepatocellular carcinoma (HCC), microvascular invasion (MVI), transcatheter arterial chemoembolization (TACE)

## Abstract

**Background:**

Early and late recurrence of hepatocellular carcinoma (HCC) have different clinical outcomes, especially for those accompanied by microvascular invasion (MVI), but the definition of early recurrence remains controversial. Therefore, a reasonable identification of the early recurrence time for HCC is urgently needed.

**Methods:**

Resected recurrence patients were enrolled and divided into two cohorts, one for identification of the early recurrence time and another for verification of the accuracy of the point. Univariable and multivariable Cox regression analyses were adopted to identify the prognostic factors of recurrence HCC (rHCC) and Kaplan–Meier method was applied to analyze the overall survival (OS). The appropriate cutoff value was determined by the exhaustive method using different recurrence intervals from 1 to 24 months in turn.

**Results:**

In total, 292 resected rHCC patients were analyzed to calculate the early recurrence interval, and another 421 resected rHCC patients with MVI were enrolled to verify the efficacy of adjuvant transarterial chemoembolization (TACE) in this recurrence interval. MVI was identified as an independent risk factor by multivariable analysis. The OS of rHCC patients without MVI is better than that of patients with MVI when the recurrence time was within 13 months, while not beyond 13 months. The verification cohort demonstrated that adjuvant TACE provided longer survival for rHCC with MVI when the recurrence time was within 13 months, while not beyond 13 months.

**Conclusion:**

For HCC patients with MVI who underwent R0 resection, 13 months may be a reasonable early recurrence time point, and within this interval, postoperative adjuvant TACE may result in longer survival compared with surgery alone.

## Graphical abstract

For hepatocellular carcinoma (HCC) patients who suffered recurrence, 13 months may be the appropriate identification for early recurrent HCC with MVI, and adjuvant TACE (transcatheter arterial chemoembolization) may give longer survival within this period.

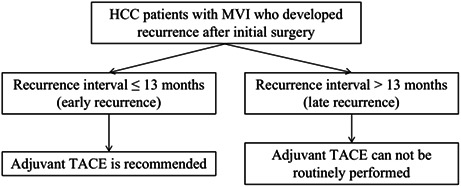

## 1 INTRODUCTION

Hepatocellular carcinoma (HCC) is the fifth most common malignancy and the third most lethal cancer worldwide.^1^ Although liver resection is recommended as the first‐line curative therapy currently,^2^, ^3^, ^4^ the survival of HCC patients is still far from satisfactory for the recurrence rate remains high with almost 70% of patients suffering from recurrence within 5 years after surgery.^5^, ^6^, ^7^ And the duration from surgery to initial recurrence is strongly associated with the prognosis of HCC patients who develop recurrence.^5^, ^8^, ^9^ Generally, early recurrence was most related to the occult micro‐metastasis derived from the initial tumor and correlated with aggressive tumor characteristics,^10^, ^11^ especially microvascular invasion (MVI)^12^, ^13^, ^14^, ^15^ which is found to be more accurate in predicting recurrence and survival outcomes than other factors included in the Milan criteria.^16^ Thus, a deeper understanding of the recurrence characteristic of HCC with MVI is important.

Several published studies have defined the early recurrence time. Some experts held the opinion that a tumor recurring within 2 years was regarded as an early recurrent tumor.^15^, ^17^ Indeed, most of these studies found that there were two peaks of recurrence in the overall recurrence curve. They determined the early recurrence time according to the appearance of the first recurrence peak at about 1–2 years without pointing out an exact one.^15^ However, most of these studies were single‐center studies, and this definition is somewhat arbitrary because it not only lacks medical statistic evidence but also disregards the feature of dominant risk factors (like MVI) during the recurrence interval. Considering these defects, a further study on the reasonable early recurrence time point of HCC patients with MVI is urgently needed for guiding clinical treatment.

In the present study, recurrence HCC (rHCC) patients who underwent a second surgery were selected, and the diagnosis of MVI was based on the initial postoperative pathological examinations. By analyzing the data of these patients, a reasonable early recurrence cutoff was determined. Besides, another contemporary adjuvant transcatheter arterial chemoembolization (TACE) cohort was enrolled to verify the accuracy and practicability of this time point to early recurrent HCC patients.

## 2 MATERIALS AND METHODS

### 2.1 Study Population

This retrospective study was conducted on consecutive HCC patients who underwent a second liver resection for rHCC between 2012 to 2016 at the Eastern Hepatobiliary Surgery Hospital of Shanghai, Chinese People's Liberation Army (PLA) General Hospital of Beijing, and Zhejiang Provincial Armed Police Corps Hospital of Hangzhou. This study was conducted in accordance with the Declaration of Helsinki (as revised in 2013) and approved by the ethical review committee of the above three participant hospitals. Informed consent was obtained from all patients before treatments. The diagnosis of rHCC was confirmed by postoperative histopathological examination. The presence of MVI was evaluated via microscopic examination of the initial entire surgical specimen and defined as a cancer cell nest within a vascular space lined by endothelium.^14^ Curative liver resection refers to the complete removal of all tumors with a microscopically clear margin (R0 resection). To avoid the effect of different tumor biology and therapeutic modalities on prognostic analysis, this study enrolled two cohorts of patients. One cohort was rHCC patients who only received a second resection to define a reasonable time point of early recurrence of HCC patients with MVI, and another cohort was MVI‐positive rHCC patients who underwent a second resection with or without adjuvant TACE to verify the accuracy of the identification. Patients who met the following were excluded^1^: diagnosed with multiple recurrent tumors or macrovascular invasion^2^; underwent palliative liver resection (R1 or R2 resection) with microscopically or grossly positive margins^3^; accepted preoperative or other concomitant antitumor regimens^4^; accompanied distant metastasis^5^; combined with refractory ascites, hepatic encephalopathy or other serious complications^6^; data regarding essential prognostic variables were missing. The study was censored on December 31, 2018.

### 2.2 Surgery and adjuvant TACE

Curative liver resection was performed via the techniques described previously.^13^, ^18^ Intraoperative ultrasonography was performed routinely which could assess the number and size of the lesions, as well as the relationship of the tumors to the surrounding vascular structures. And when Pringle's maneuver was conducted, a clamp/unclamp time of 10 min/5 min should be complied. R0 anatomical resection was considered as the curative liver resection for a single tumor. For multiple bilobar tumor nodules, the main tumor was resected anatomically, while satellite nodules were resected non‐anatomically with an adequate resection margin. If the liver remnant volume in patients was inadequate, non‐anatomical resection was adopted to achieve a negative resection margin. A negative resection margin was defined as a lack of visible tumor cells left in the remnant liver at all the resection margins.

Adjuvant TACE was performed at 4 weeks after liver surgery. Using the Seldinger technique, the catheter was operated into the proper hepatic artery through the femoral artery. Any obvious tumor stains in the remnant liver were detected via Hepatic angiography, CT angiography, or both. And then pirarubicin (THP) or pharmorubicin (20–40 mg), doxorubicin hydrochloride (10 mg), and lipiodol (2–10 mL) were infused into the catheter. Body surface area and underlying liver function should be calculated to determine the accurate dosage of lipiodol and doxorubicin. The efficacy of TACE was evaluated by a CT scan after 1 month.

### 2.3 Follow‐up

After hospital discharge, patients' postoperative surveillance and management protocols were uniformly formulated. Physical examination, serum alpha‐fetoprotein (AFP) level, and imaging examination (including ultrasonography, contrast‐enhanced computed tomography (CT) scan, or magnetic resonance imaging (MRI)) were investigated once every 2 months for the first 6 months after liver surgery and then the frequency would be once every 3 months for the subsequent 1.5 years. If no recurrence occurred after 2 years of resection, patients would be followed up every 6 months.

Once HCC recurrence was suspected during the follow‐up period, contrast‐enhanced CT or MRI was conducted, and then further examinations were carried out, including positron emission tomography‐CT (PET‐CT), full‐body bone scan, or angiography. The diagnosis of rHCC was based on clinical investigations or the result of a histological biopsy. Details of the diagnosis, interval to recurrence, presence of MVI, treatment for recurrence, and overall survival (OS) were assessed to investigate the association between the recurrence time and MVI. The duration from the second liver resection to either death or the last follow‐up was calculated as OS.

### 2.4 The early recurrence identification procedure

**FIGURE 1:**
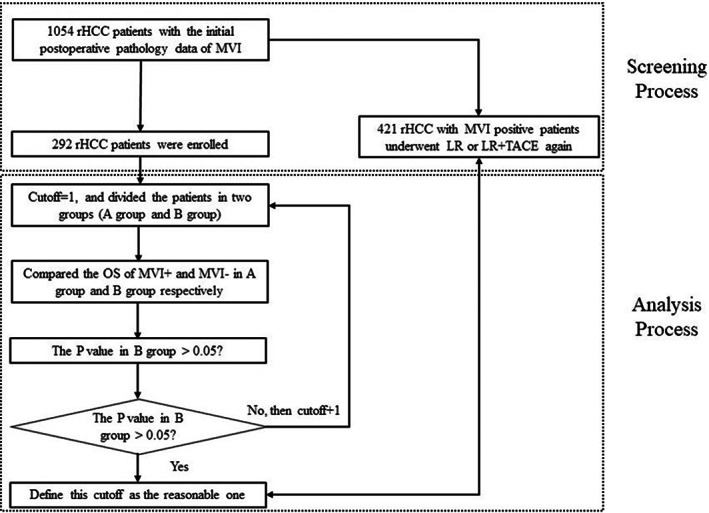
The flowchart to select eligible recurrence HCC patients and the analysis process for the study. A group, rHCC patients whose recurrence time is shorter than cutoff time; B group, rHCC patients whose recurrence time is longer than cutoff time. Abbreviations: LR, liver resection; MVI, microvascular invasion; OS, overall survival; rHCC, recurrence hepatocellular carcinoma; TACE, transcatheter arterial chemoembolization.

The exhaustive method was applied to define the reasonable time point of early recurrence of HCC associated with MVI (Figure 1). The cutoff time was adopted gradually from 1 month to 24 months, and the interval between each cutoff time is 1 month. When each cutoff time was adopted, the enrolled patients could be divided into two groups: recurrence time within the cutoff time (Group A) and beyond the cutoff time (Group B). The survival difference between patients with or without MVI was compared in Groups A and B, respectively. When the *p* < 0.05 in Group A but >0.05 in Group B, the cutoff was considered the optimal time point for MVI‐positive patients.

### 2.5 Statistical Analysis

Continuous variables were analyzed by Student's *t*‐test and categorical variables were analyzed by *χ* ^2^ or Fisher's exact test. Survival curves were determined by the Kaplan–Meier method. Univariate analysis was analyzed by the log‐rank test. Cox proportional hazards regression model was used for multivariate analysis to assess prognostic factors that were significant in the univariate analysis. The prognostic factors incorporated into multivariate analysis were those with a *p* value <0.05 on univariate analysis. Two‐tailed *p* < 0.05 was considered statistically significant.

## 3 RESULTS

### 3.1 Patient population

In total, 292 of 1054 rHCC patients who underwent open curative liver resection for a single recurrent tumor were included to analyze the reasonable identification of early recurrence based on MVI (Figure 1). The patients' characteristics are shown in Table 1. Most patients were HBV‐related (92.1%) and Child‐Pugh A class (99.3%). Table 2 showed that MVI was one of the most important risk factors for rHCC. And there was almost no difference in clinicopathological variables between the MVI positive and MVI negative groups **(**Table S1). Figure S1 showed that the OS of the MVI negative group was significantly longer than the MVI positive group. The OS rates of 1‐, 3‐, and 5‐year were 97.1%, 87.4%, and 77.2% in the MVI negative group, while 80.5%, 59.2%, and 52.9% in the MVI positive group, respectively.

**TABLE 1:** Baseline patient demographics.

Number of patients	292
Age at treatment, median (range)	50.5 years (20.0–72.0 years)
% Male	263/292 (90.1%)
WBC (×10^6^/L)	
≤4000	61 (20.9%)
>4000	231 (79.1%)
RBC (×10^12^/L)	
≤4	21
>4	271
PLT (×10^9^/L)	
≤100	57
>100	235
PT (s)	
≤13	247
>13	45
TBil (μmol/L)	
≤17.1	227
>17.1	65
ALB (g/L)	
≤40	87
>40	205
ALT (U/L)	
≤40	176
>40	116
AST (U/L)	
≤35	168
>35	124
GGT (U/L)	
≤50	84
>50	208
ALP (U/L)	
≤150	275
>150	17
AFP (ng/mL)	
≤400	201
>400	91
HBsAg	
positive	269
negative	23
HBsAb	
Positive	230
Negative	62
Child‐Pugh class	
A	290
B	2
Tumor diameter (cm)	
≤5	220
>5	72

Abbreviations: AFP, alpha‐fetoprotein; ALB, albumin; ALP, alkaline phosphatase; ALT, alanine aminotransferase; AST, aspartate aminotransferase; GGT, gamma‐glutamyl‐transferase; HBsAb, hepatitis B surface antibody; HBsAg, hepatitis B surface antigen; HCC, hepatocellular carcinoma; MVI, microvascular invasion; PLT, platelet; PT, prothrombin time; RBC, red blood cell; TBil, total bilirubin; WBC, white blood cell.

**TABLE 2:** Univariate and multivariate analysis for prognostic factors of OS.

Clinical variables	Univariate analysis		Multivariate analysis	
	HR (95% CI)	*p*	HR (95% CI)	*p*
MVI, positive	2.63 (1.71, 4.05)	<0.001	2.33 (1.49, 3.66)	<0.001
Age >55 years	1.28 (0.82, 1.99)	0.279		
Sex, male	1.37 (0.63, 2.97)	0.424		
WBC, >4000 × 10^6^/L	1.18 (0.69, 2.00)	0.551		
RBC, ≤4 × 10^12^/L	1.16 (0.51, 2.66)	0.729		
PLT, ≤100 × 10^9^/L	0.78 (0.45, 1.35)	0.378		
PT >13 s	1.60 (0.94, 2.71)	0.085		
TBil >17.1 μmol/L	0.94 (0.55, 1.60)	0.826		
ALB ≤40 g/L	1.71 (1.11, 2.64)	0.016		
ALT >40 U/L	1.28 (0.84, 1.95)	0.259		
AST > 35 U/L	2.27 (1.48, 3.48)	<0.001		
GGT > 50 IU/L	1.71 (1.04, 2.83)	0.035		
ALP > 150 IU/L	7.08 (3.87, 12.93)	<0.001	2.74 (1.43, 5.25)	0.002
AFP > 400 ng/mL	1.67 (1.08, 2.58)	0.021		
HBsAg, positive	1.00 (0.44, 2.31)	0.993		
HBsAb, positive	2.30 (1.46, 3.62)	<0.001	2.23 (1.38, 3.58)	0.001
Tumor diameter, >5 cm	5.43 (3.36, 8.76)	<0.001	4.25 (2.49, 7.23)	<0.001

Abbreviations: AFP, alpha‐fetoprotein; ALB, albumin; ALP, alkaline phosphatase; ALT, alanine aminotransferase; AST, aspartate aminotransferase; GGT, gamma‐glutamyl‐transferase; HBsAb, hepatitis B surface antibody; HBsAg, hepatitis B surface antigen; HCC, hepatocellular carcinoma; MVI, microvascular invasion; PLT, platelet; PT, prothrombin time; RBC, red blood cell; TBil, total bilirubin; WBC, white blood cell.

**FIGURE 2:**
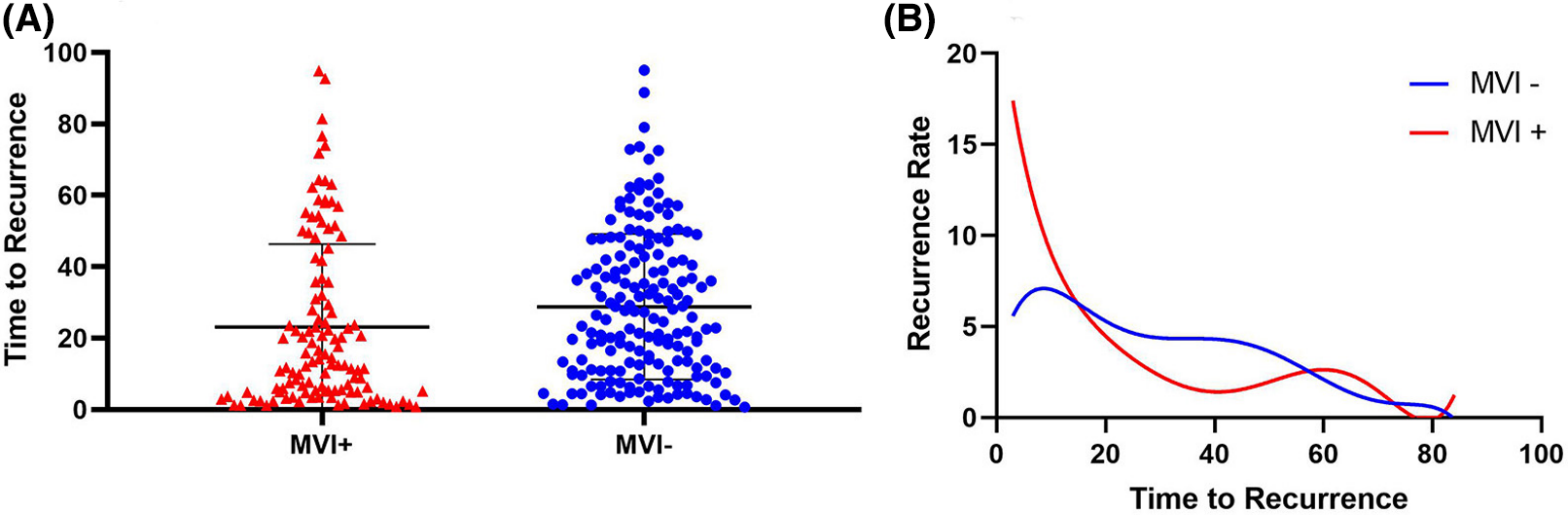
The regulation of rHCC with or without MVI. (A) All recurrence interval data of each enrolled patients were depicted in the nested; (B) The recurrence rate curve of various postoperative follow‐up time. Abbreviation: rHCC, recurrent hepatocellular carcinoma.

The recurrence rate curves in different recurrence intervals were depicted (Figure 2). Of note, for the MVI‐positive group, there were two peaks of recurrence where the first occurred around 1–2 years after initial surgical resection, and the second appeared about 5 years after surgery. For the MVI‐negative group, the incidence of recurrence decreased gradually over time.

### 3.2 Different cutoff time of recurrence

**FIGURE 3:**
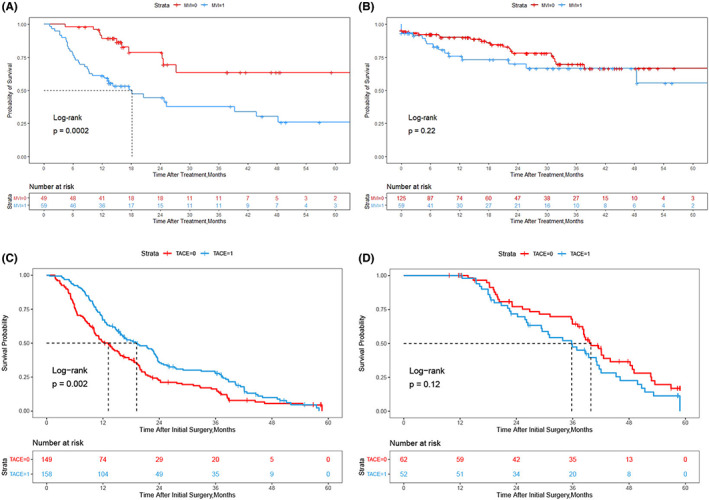
Kaplan–Meier survival curves of OS in rHCC patients with or without MVI in 13 months recurrence interval (A) and beyond 13 months recurrence interval (B). Kaplan–Meier survival curves of OS in MVI positive rHCC patients underwent adjuvant TACE or not in 13 months recurrence interval (C) and beyond 13 months recurrence interval (D). Abbreviations: OS, overall survival; rHCC, recurrent hepatocellular carcinoma; MVI, microvascular invasion; TACE, transcatheter arterial chemoembolization.

When the cutoff time is chosen at 13 months after the first resection, the OS of patients without MVI is not significantly longer than that of patients with MVI(Table S2). Tables S3 and S4 show that there was almost no difference in the clinicopathological variables between MVI‐positive patients and MVI‐negative patients whose recurrence time was shorter or longer than 13 months. And the OS of MVI‐negative patients was significantly longer than that of MVI‐positive patients if the recurrence time was shorter than 13 months (Figure 3A), while there was no significant difference if the recurrence time was longer than 13 months (Figure 3B).

### 3.3 The clinical value of the cutoff time at 13 months for early recurrence

To validate the accuracy and practicability of 13 months to differentiate early and late recurrence, we selected another 421 of 1054 rHCC patients with MVI who underwent surgical resection with or without adjuvant TACE (Figure 1). The clinicopathological variables between the LR + TACE group and the LR group in shorter recurrence time part (recurrence time shorter than 13 months) (Table S5) and longer recurrence time part (recurrence time longer than 13 months) (Table S6) were almost comparable. In shorter recurrence time part, patients in the LR + TACE group had markedly OS than the LR group (median OS 19.2 vs. 13.1 months, *p* = 0.002; Figure 3C). In longer recurrence time part, patients in the LR + TACE group had similar OS compared with the LR group (median OS 39.9 vs. 35.8 months, *p* = 0.12; Figure 3D).

## 4 DISCUSSION

The recurrence of HCC after surgical resection, especially early recurrence, has been plagued surgeons for a long time. HCC recurrence represents the intrahepatic occult metastasis originating from the initial tumor or de novo tumors, which were defined as the early recurrence or late recurrence respectively.^19^ Identification of a reasonable time intercept point of early recurrence and then appropriate treatment of the early rHCC is important for promoting long‐term survival outcomes of rHCC patients. Our study identified the reasonable cutoff of the early recurrence time was 13 months for HCC with MVI after R0 resection, which indicated that MVI was one of the most critical factors for recurrence during this period. Based on this identification, another cohort was enrolled to compare the different effects of postoperative adjuvant TACE during the recurrence interval, and the result confirmed the accuracy of this identification as well as the importance of adjuvant TACE for early recurrence HCC with MVI.

To our knowledge, this research is the first multicenter study that defines 13 months as the reasonable early recurrence time with the aspect of MVI. Briefly, the outcome events and survival time of MVI positive and negative were taken into consideration and were compared in various recurrence time groups respectively. Using the exhaustive method to analyze the *p*‐value,^20^ the result of our study showed that 13 months should be more statistical and reasonable to reflect the feature of MVI in early recurrence.^21^ In contrast with other studies generally using 2 years as the cutoff value for early recurrence in HCC,^22^, ^23^, ^24^ using 13 months could predict early recurrence and survival outcomes more accurately in MVI‐positive patients,^25^, ^26^ and also demonstrates that the positive postoperative adjuvant treatments are recommended in the early recurrence interval.

Considering the valuable MVI data could predict HCC recurrence more accurately,^27^ we worked to decipher the appropriate early recurrence identification with respect to MVI. The general definition of early recurrence is derived from the result of appraising recurrence rate regulation which excludes the dominant risk factors and may cause the contradiction of accuracy in different studies' results. In this study, via multivariable Cox regression analyses, MVI resulted in a poor long‐term prognosis of rHCC which was similar to the previous studies.^14^, ^17^, ^28^, ^29^, ^30^ Compared with other risk factors, exploring the relationship between MVI and recurrence regulation may be more helpful for decision‐making about treatment options for early recurrence.^12^, ^18^, ^31^, ^32^ Because the presence of MVI represents the breakdown of surrounding extracellular matrix (ECM), loss of cell–cell adhesion, more use of alternative energy sources, and more cellular motility.^33^, ^34^, ^35^, ^36^ All these alterations generate the carcinogenic microenvironment in the liver^37^ and provoke the invasion of HCC into either the luminal structure or hepatic parenchyma.^16^ In other words, MVI had a much closer relationship with early recurrence than other factors such as ALP and HBsAb, implying that the earlier HCC recurred, the more effect MVI produces, and more efficacy intervention treatments for MVI may possess.

The wider use of semi‐annual surveillance expands the proportion of recurrent tumors that qualified for surgical resection. And thanks to the evolution of minimally invasive surgical techniques and laparoscopic liver resection, more patients could tolerate a radical surgery, including some selected Child‐Pugh B class patients.^38^ Besides, the development of loco‐regional treatment after resection, like adjuvant TACE, further improved the outcome of rHCC patients.^39^ Therefore, we enrolled the rHCC with MVI patients who underwent adjuvant TACE or not after a second resection to verify the accuracy of using 13 months as the appropriate identification for early recurrence. The result demonstrated that adjuvant TACE was efficacious in the early recurrence interval. Adjuvant therapy is recommended for preventing intrahepatic microscopic metastasis,^40^ but some current studies are still controversial for the efficacy of the combination of adjuvant TACE in MVI‐positive patients. Our previous study estimated the impact of adjuvant TACE among 322 MVI‐positive patients, and both the OS and RFS (recurrence‐free survival) in the postoperative adjuvant TACE group were significantly improved.^13^ In contrast, Wang et al. observed adjuvant TACE could only significantly prolong the DFS (disease‐free survival) and OS for MVI‐positive patients beyond the Milan criteria.^41^ Combining the result of our study, we postulate the controversy between different research may be interpreted by respective roles of MVI at different recurrence periods, which means adjuvant therapy for MVI is effective in the early recurrence stage while futile after the early recurrence stage.

The present study may be limited by the following fields. First, inherent biases are inevitable due to the retrospective nature of this study. Second, to simplify the study process and obtain the unprecedented time definition of early recurrence, we only enrolled the rHCC patients who underwent surgery again so as to eliminate the interference of different treatment methods for the long‐term prognosis. And it is indeed hard to take other treatments into consideration except surgery for us. Third, MVI is diagnosed by the evaluation of tumor and surrounding hepatic tissues which is accomplished by pathologists, the false positive or false negative may exist.

## 5 CONCLUSION

The recurrent interval of 13 months may be a reasonable early recurrence cutoff value for those rHCC patients with MVI, and active treatments, such as TACE, for MVI are recommended when tumor recurrence occurs within 13 months. And this work provides a foundation for further research on the efficacy of different treatments for rHCC.

## AUTHOR CONTRIBUTIONS

**Zonghan Liu:** Conceptualization (equal); data curation (equal); formal analysis (equal); investigation (equal); methodology (equal); software (equal); visualization (equal); writing – original draft (equal); writing – review and editing (equal). **Zongtao Chai:** Conceptualization (equal); data curation (equal); formal analysis (equal); funding acquisition (equal); methodology (equal); project administration (equal); supervision (equal); writing – review and editing (equal). **Jin‐kai Feng:** Conceptualization (equal); data curation (equal); formal analysis (equal); software (equal); writing – original draft (supporting). **Yuchao Hou:** Data curation (equal); formal analysis (equal); investigation (equal). **Xiu‐Ping Zhang:** Data curation (equal); formal analysis (equal); resources (equal). **Zhen‐Hua Chen:** Data curation (equal); methodology (supporting); resources (equal). **Yan‐Jun Xiang:** Investigation (supporting); methodology (supporting). **Wei‐Xing Guo:** Resources (equal); validation (equal). **Jie Shi:** Resources (equal); validation (equal). **Shuqun Cheng:** Conceptualization (equal); funding acquisition (equal); methodology (equal); project administration (equal); resources (equal); supervision (equal); validation (equal); writing – original draft (equal); writing – review and editing (equal).

## FUNDING INFORMATION

This work was supported by the Clinical Research Plan of Shanghai Hospital Development Center (SHDC2020CR1004A), the Key Project of the National Natural Science Foundation of China (81730097), the National Natural Science Foundation of China (82072618), the National Natural Science Foundation of China (82172846), and the National Key Research and Development Program of China (2022YFC2503700).

## CONFLICT OF INTEREST STATEMENT

All participated authors have no conflicts of interest related to this study.

## ETHICS STATEMENT

This study was complied with the declaration of Helsinki and the Clinical Research Ethics Committee of Eastern Hepatobiliary Surgery Hospital (EHBH), Chinese People's Liberation Army (PLA) General Hospital, and Zhejiang Provincial Armed Police Corps Hospital approved this retrospective study. No personal information in this study was disclosed.

## Supporting information


Figure S1

Table S1

Table S2

Table S3

Table S4

Table S5

Table S6

## Data Availability

The raw data supporting the conclusions of the study are available by the corresponding author, without undue reservation.
