# Supplementary figures and images for: A reasonable identification of the early recurrence time based on microvascular invasion for hepatocellular carcinoma after R0 resection: A multicenter retrospective study
Source: Cancer Med. 2023 Mar 6;12(9):10294–302. doi: 10.1002/cam4.5758 (PMC10225226; doi:10.1002/cam4.5758)

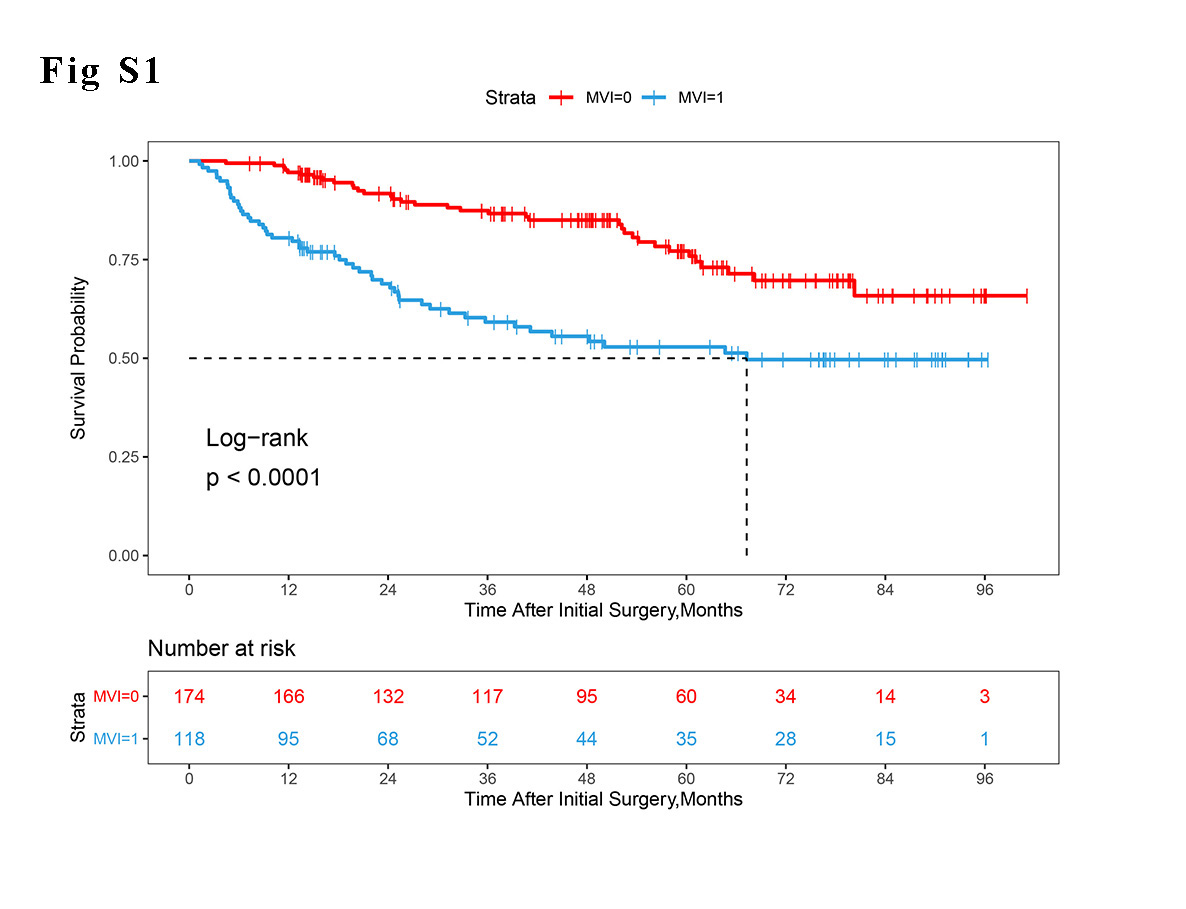

Supplement: Supplementary file 1 — Figure S1 [file CAM4-12-10294-s006.jpg]
