# Supplementary material for: A reasonable identification of the early recurrence time based on microvascular invasion for hepatocellular carcinoma after R0 resection: A multicenter retrospective study
Source: Cancer Med. 2023 Mar 6;12(9):10294–302. doi: 10.1002/cam4.5758 (PMC10225226; doi:10.1002/cam4.5758)
Supplement: Supplementary file 2 — Table S1 [file CAM4-12-10294-s002.docx]

| **Table S1.** Baseline characteristics of HCC patients with MVI or without MVI | | | |
| --- | --- | --- | --- |
| **Variables** | **MVI Positive**  **(n=118)** | **MVI Negative**  **(n=174)** | ***P*** |
| Age (year) |  |  | 0.415 |
| ≤ 55 | 84 (71.2%) | 116 (66.7%) |  |
| > 55 | 34 (28.8%) | 58 (33.3%) |  |
| Sex |  |  | 0.610 |
| Male | 105 (89.0%) | 158 (90.8%) |  |
| Female | 13 (11.0%) | 16 (9.2%) |  |
| WBC (*10^6^/L) |  |  | 0.849 |
| ≤ 4000 | 24 (20.3%) | 37 (21.3%) |  |
| > 4000 | 94 (79.7%) | 137 (78.7%) |  |
| RBC (*10^12^/L) |  |  | 0.251 |
| ≤ 4 | 6 (5.1%) | 15 (8.6%) |  |
| > 4 | 112 (94.9%) | 159 (91.4%) |  |
| PLT (*10^9^/L) |  |  | 0.756 |
| ≤ 100 | 22 (18.6%) | 35 (20.1%) |  |
| > 100 | 96 (81.4%) | 139 (79.9%) |  |
| PT (s) |  |  | 0.352 |
| ≤ 13 | 97 (82.2%) | 150 (86.2%) |  |
| > 13 | 21 (17.8%) | 24 (13.8%) |  |
| TBil (μmol/L) |  |  | 0.433 |
| ≤ 17.1 | 89 (75.4%) | 138 (79.3%) |  |
| > 17.1 | 29 (24.6%) | 36 (20.7%) |  |
| ALB (g/L) |  |  | 0.826 |
| ≤ 40 | 36 (30.5%) | 51 (29.3%) |  |
| > 40 | 82 (69.5%) | 123 (70.7%) |  |
| ALT (U/L) |  |  | 0.315 |
| ≤ 40 | 67 (56.8%) | 109 (62.6%) |  |
| > 40 | 51 (43.2%) | 65 (37.4%) |  |
| AST (U/L) |  |  | 0.096 |
| ≤ 35 | 61 (51.7%) | 107 (61.5%) |  |
| > 35 | 57 (48.3%) | 67 (38.5%) |  |
| GGT (U/L) |  |  | 0.018 |
| ≤ 50 | 25 (21.2%) | 59 (33.9%) |  |
| > 50 | 93 (78.8%) | 115 (66.1%) |  |
| ALP (U/L) |  |  | 0.009 |
| ≤ 150 | 106 (89.8%) | 169 (97.1%) |  |
| > 150 | 12 (10.2%) | 5 (2.9%) |  |
| AFP (ng/mL) |  |  | < 0.001 |
| ≤ 400 | 63 (53.4%) | 138 (79.3%) |  |
| > 400 | 55 (46.6%) | 36 (20.7%) |  |
| HBsAg |  |  | 0.567 |
| Positive | 110 (93.2%) | 159 (91.4%) |  |
| Negative | 8 (6.8%) | 15 (8.6%) |  |
| HBsAb |  |  | 0.083 |
| Negative | 87 (73.7%) | 143 (82.2%) |  |
| Positive | 31 (26.3%) | 31 (17.8%) |  |
| Child-Pugh class |  |  | 0.162 |
| A | 116 (98.3%) | 174 (100.0%) |  |
| B | 2 (1.7%) | 0 (0.0%) |  |
| Tumor diameter (cm) |  |  | 0.175 |
| ≤ 5 | 84 (71.2%) | 136 (78.2%) |  |
| > 5 | 34 (28.8%) | 38 (21.8%) |  |
| Note: HCC, hepatocellular carcinoma; MVI, microvascular invasion; WBC, white blood cell; RBC, red blood cell; PLT, platelet; PT, prothrombin time; TBil, total bilirubin; ALB, albumin; ALT, alanine aminotransferase; AST, aspartate aminotransferase; GGT, gamma-glutamyl-transferase; ALP, alkaline phosphatase; AFP, alpha-fetoprotein; HBsAg, hepatitis B surface antigen; HBsAb, hepatitis B surface antibody | | | |
|  | | | |
