# Supplementary material for: A reasonable identification of the early recurrence time based on microvascular invasion for hepatocellular carcinoma after R0 resection: A multicenter retrospective study
Source: Cancer Med. 2023 Mar 6;12(9):10294–302. doi: 10.1002/cam4.5758 (PMC10225226; doi:10.1002/cam4.5758)
Supplement: Supplementary file 3 — Table S2 [file CAM4-12-10294-s001.docx]

| **Table S2.** P-value for the comparison of the OS of MVI positive and MVI negative | | |
| --- | --- | --- |
| Cutoff value (month) | BCTg (recurrence time shorter than cutoff value) | ACTg (recurrence time longer than cutoff value) |
| 1 | 0.029 | 0.000 |
| 2 | 0.099 | 0.000 |
| 3 | 0.083 | 0.000 |
| 4 | 0.033 | 0.000 |
| 5 | 0.003 | 0.000 |
| 6 | 0.005 | 0.004 |
| 7 | 0.001 | 0.011 |
| 8 | 0.002 | 0.006 |
| 9 | 0.001 | 0.024 |
| 10 | 0.001 | 0.018 |
| 11 | 0.000 | 0.030 |
| 12 | 0.000 | 0.041 |
| 13 | 0.000 | 0.122 |
| 14 | 0.000 | 0.138 |
| 15 | 0.000 | 0.277 |
| 16 | 0.000 | 0.363 |
| 17 | 0.000 | 0.297 |
| 18 | 0.000 | 0.300 |
| 19 | 0.000 | 0.334 |
| 20 | 0.000 | 0.332 |
| 21 | 0.000 | 0.820 |
| 22 | 0.000 | 0.736 |
| 23 | 0.000 | 0.853 |
| 24 | 0.000 | 0.410 |
| Note: OS, overall survival; MVI, microvascular invasion; BCTg, before cutoff time group; ACTg, after cutoff time group | | |
