# Supplementary material for: A reasonable identification of the early recurrence time based on microvascular invasion for hepatocellular carcinoma after R0 resection: A multicenter retrospective study
Source: Cancer Med. 2023 Mar 6;12(9):10294–302. doi: 10.1002/cam4.5758 (PMC10225226; doi:10.1002/cam4.5758)
Supplement: Supplementary file 4 — Table S3 [file CAM4-12-10294-s003.docx]

| **Table S3.** Baseline characteristics of HCC patients whose recurrence time shorter than 13 months | | | |
| --- | --- | --- | --- |
| **Variables** | **MVI Positive**  **(n=59)** | **MVI Negative**  **(n=49)** | ***P*** |
| Age (year) |  |  | 0.408 |
| ≤ 55 | 44 (74.6%) | 33 (67.3%) |  |
| > 55 | 15 (25.4%) | 16 (32.7%) |  |
| Sex |  |  | 1.000 |
| Male | 53 (89.8%) | 45 (91.8%) |  |
| Female | 6 (10.2%) | 4 (8.2%) |  |
| WBC (*10^6^/L) |  |  | 0.145 |
| ≤ 4000 | 8 (13.6%) | 12 (24.5%) |  |
| > 4000 | 51 (86.4%) | 37 (75.5%) |  |
| RBC (*10^12^/L) |  |  | 0.220 |
| ≤ 4 | 4 (6.8%) | 7 (14.3%) |  |
| > 4 | 55 (93.2%) | 42 (85.7%) |  |
| PLT (*10^9^/L) |  |  | 0.495 |
| ≤ 100 | 8 (13.6%) | 9 (18.4%) |  |
| > 100 | 51 (86.4%) | 40 (81.6%) |  |
| PT (s) |  |  | 0.818 |
| ≤ 13 | 48 (81.4%) | 39 (79.6%) |  |
| > 13 | 11 (18.6%) | 10 (20.4%) |  |
| TBil (μmol/L) |  |  | 0.460 |
| ≤ 17.1 | 48 (81.4%) | 37 (75.5%) |  |
| > 17.1 | 11 (18.6%) | 12 (24.5%) |  |
| ALB (g/L) |  |  | 0.733 |
| ≤ 40 | 21 (35.6%) | 19 (38.8%) |  |
| > 40 | 38 (64.4%) | 30 (61.2%) |  |
| ALT (U/L) |  |  | 0.280 |
| ≤ 40 | 30 (50.8%) | 30 (61.2%) |  |
| > 40 | 29 (49.2%) | 19 (38.8%) |  |
| AST (U/L) |  |  | 0.027 |
| ≤ 35 | 20 (33.9%) | 27 (55.1%) |  |
| > 35 | 39 (66.1%) | 22 (44.9%) |  |
| GGT (U/L) |  |  | 0.023 |
| ≤ 50 | 5 (8.5%) | 12 (24.5%) |  |
| > 50 | 54 (91.5%) | 37 (75.5%) |  |
| ALP (U/L) |  |  | 0.005 |
| ≤ 150 | 44 (80.0%) | 47 (97.9%) |  |
| > 150 | 11 (20.0%) | 1 (2.1%) |  |
| AFP (ng/mL) |  |  | 0.002 |
| ≤ 400 | 26 (44.1%) | 36 (73.5%) |  |
| > 400 | 33 (55.9%) | 13 (26.5%) |  |
| HBsAg |  |  | 0.500 |
| Positive | 57 (96.6%) | 49 (100.0%) |  |
| Negative | 2 (3.4%) | 0 (0.0%) |  |
| HBsAb |  |  | 0.286 |
| Positive | 20 (33.9%) | 12 (24.5%) |  |
| Negative | 39 (66.1%) | 37 (75.5%) |  |
| Tumor diameter (cm) |  |  | 0.562 |
| ≤ 5 | 28 (47.5%) | 26 (53.1%) |  |
| > 5 | 31 (57.4%) | 23 (46.9%) |  |
| Note: HCC, hepatocellular carcinoma; MVI, microvascular invasion; WBC, white blood cell; RBC, red blood cell; PLT, platelet; PT, prothrombin time; TBil, total bilirubin; ALB, albumin; ALT, alanine aminotransferase; AST, aspartate aminotransferase; GGT, gamma-glutamyl-transferase; ALP, alkaline phosphatase; AFP, alpha-fetoprotein; HBsAg, hepatitis B surface antigen; HBsAb, hepatitis B surface antibody | | | |
|  | | | |
