# Supplementary material for: A reasonable identification of the early recurrence time based on microvascular invasion for hepatocellular carcinoma after R0 resection: A multicenter retrospective study
Source: Cancer Med. 2023 Mar 6;12(9):10294–302. doi: 10.1002/cam4.5758 (PMC10225226; doi:10.1002/cam4.5758)
Supplement: Supplementary file 5 — Table S4 [file CAM4-12-10294-s007.docx]

| **Table S4.** Baseline characteristics of HCC patients whose recurrence time longer than 13 months | | | |
| --- | --- | --- | --- |
| **Variables** | **MVI Positive**  **(n=59)** | **MVI Negative**  **(n=125)** | ***P*** |
| Age (year) |  |  | 0.851 |
| ≤ 55 | 40 (67.8%) | 83 (66.4%) |  |
| > 55 | 19 (32.2%) | 42 (33.6%) |  |
| Sex |  |  | 0.638 |
| Male | 52 (88.1%) | 113 (90.4%) |  |
| Female | 7 (11.9%) | 12 (9.6%) |  |
| WBC (*10^6^/L) |  |  | 0.279 |
| ≤ 4000 | 16 (27.1%) | 25 (20.0%) |  |
| > 4000 | 43 (72.9%) | 100 (80.0%) |  |
| RBC (*10^12^/L) |  |  | 0.505 |
| ≤ 4 | 2 (3.4%) | 8 (6.4%) |  |
| > 4 | 57 (96.6%) | 117 (93.6%) |  |
| PLT (*10^9^/L) |  |  | 0.653 |
| ≤ 100 | 14 (23.7%) | 26 (20.8%) |  |
| > 100 | 45 (76.3%) | 99 (79.2%) |  |
| PT (s) |  |  | 0.280 |
| ≤ 13 | 49 (83.1%) | 111 (88.8%) |  |
| > 13 | 10 (16.9%) | 14 (11.2%) |  |
| TBil (μmol/L) |  |  | 0.088 |
| ≤ 17.1 | 41 (69.5%) | 101 (80.8%) |  |
| > 17.1 | 18 (30.5%) | 24 (19.2%) |  |
| ALB (g/L) |  |  | 0.980 |
| ≤ 40 | 15 (25.4%) | 32 (25.6%) |  |
| > 40 | 44 (74.6%) | 93 (74.4%) |  |
| ALT (U/L) |  |  | 0.949 |
| ≤ 40 | 37 (62.7%) | 79 (63.2%) |  |
| > 40 | 22 (37.3%) | 46 (36.8%) |  |
| AST (U/L) |  |  | 0.464 |
| ≤ 35 | 41 (69.5%) | 80 (64.0%) |  |
| > 35 | 18 (30.5%) | 45 (36.0%) |  |
| GGT (U/L) |  |  | 0.626 |
| ≤ 50 | 20 (33.9%) | 47 (37.6%) |  |
| > 50 | 39 (66.1%) | 78 (62.4%) |  |
| ALP (U/L) |  |  | 1.000 |
| ≤ 150 | 58 (98.3%) | 121 (96.8%) |  |
| > 150 | 1 (1.7%) | 4 (3.2%) |  |
| AFP (ng/mL) |  |  | 0.005 |
| ≤ 400 | 37 (62.7%) | 102 (81.6%) |  |
| > 400 | 22 (37.3%) | 23 (18.4%) |  |
| HBsAg |  |  | 0.716 |
| Positive | 53 (89.8%) | 110 (88.0%) |  |
| Negative | 6 (10.2%) | 15 (12.0%) |  |
| HBsAb |  |  | 0.555 |
| Positive | 11 (18.6%) | 19 (15.2%) |  |
| Negative | 48 (81.4%) | 106 (84.8%) |  |
| Tumor diameter (cm) |  |  | 0.141 |
| ≤ 5 | 56 (94.9%) | 110 (88.0%) |  |
| > 5 | 3 (5.1%) | 15 (12.0%) |  |
| Note: HCC, hepatocellular carcinoma; MVI, microvascular invasion; WBC, white blood cell; RBC, red blood cell; PLT, platelet; PT, prothrombin time; TBil, total bilirubin; ALB, albumin; ALT, alanine aminotransferase; AST, aspartate aminotransferase; GGT, gamma-glutamyl-transferase; ALP, alkaline phosphatase; AFP, alpha-fetoprotein; HBsAg, hepatitis B surface antigen; HBsAb, hepatitis B surface antibody | | | |
|  | | | |
