# Supplementary material for: A reasonable identification of the early recurrence time based on microvascular invasion for hepatocellular carcinoma after R0 resection: A multicenter retrospective study
Source: Cancer Med. 2023 Mar 6;12(9):10294–302. doi: 10.1002/cam4.5758 (PMC10225226; doi:10.1002/cam4.5758)
Supplement: Supplementary file 6 — Table S5 [file CAM4-12-10294-s005.docx]

| **Table S5.** Baseline characteristics of short-term recurrent HCC patients with MVI | | | |
| --- | --- | --- | --- |
| **Variables** | **LR+TACE**  **(n=158)** | **LR**  **(n=149)** | ***P*** |
| Age (year) |  |  | 0.143 |
| ≤ 55 | 120 (75.9%) | 102 (68.5%) |  |
| > 55 | 38 (24.1%) | 47 (31.5%) |  |
| Sex |  |  | 0.584 |
| Male | 143 (90.5%) | 132 (88.6%) |  |
| Female | 15 (9.5%) | 17 (11.4%) |  |
| WBC (*10^6^/L) |  |  | 0.011 |
| ≤ 4000 | 20 (12.7%) | 37 (24.8%) |  |
| > 4000 | 138 (87.3%) | 112 (75.2%) |  |
| RBC (*10^12^/L) |  |  | 0.287 |
| ≤ 4 | 16 (10.1%) | 21 (14.1%) |  |
| > 4 | 142 (89.9%) | 128 (85.9%) |  |
| PLT (*10^9^/L) |  |  | 0.088 |
| ≤ 100 | 24 (15.2%) | 34 (22.8%) |  |
| > 100 | 134(84.8%) | 115 (77.2%) |  |
| PT (s) |  |  | 0.478 |
| ≤ 13 | 141(89.2%) | 117 (78.5%) |  |
| > 13 | 17 (10.8%) | 32 (21.5%) |  |
| TBil (μmol/L) |  |  | 0.106 |
| ≤ 17.1 | 126 (79.7%) | 102 (68.5%) |  |
| > 17.1 | 32 (20.3%) | 47 (31.5%) |  |
| ALB (g/L) |  |  | 0.327 |
| ≤ 40 | 51 (32.3%) | 56 (37.6%) |  |
| > 40 | 107 (67.7%) | 93 (62.4%) |  |
| ALT (U/L) |  |  | 0.360 |
| ≤ 40 | 70 (44.3%) | 71 (47.7%) |  |
| > 40 | 88 (55.7%) | 78 (52.3%) |  |
| AFP (ng/mL) |  |  | 0.445 |
| ≤ 400 | 76 (48.1%) | 64 (43.0%) |  |
| > 400 | 82 (51.9%) | 85 (57.0%) |  |
| HBsAg |  |  | 0.386 |
| Positive | 139 (88.0%) | 125 (83.9%) |  |
| Negative | 19 (12.0%) | 24 (16.1%) |  |
| HBsAb |  |  | 0.453 |
| Positive | 13 (8.2%) | 16 (10.7%) |  |
| Negative | 145 (91.8%) | 133 (89.3%) |  |
| Child-Pugh class |  |  | 0.065 |
| A | 155 (98.1%) | 142 (95.3%) |  |
| B | 3 (1.9%) | 7 (4.7%) |  |
| Tumor diameter (cm) |  |  | 0.167 |
| ≤ 5 | 47 (29.7%) | 62 (41.6%) |  |
| > 5 | 111 (70.3%) | 87 (58.4%) |  |
| Note: HCC, hepatocellular carcinoma; MVI, microvascular invasion; WBC, white blood cell; RBC, red blood cell; PLT, platelet; PT, prothrombin time; TBil, total bilirubin; ALB, albumin; ALT, alanine aminotransferase; AFP, alpha-fetoprotein; HBsAg, hepatitis B surface antigen; HBsAb, hepatitis B surface antibody | | | |
|  | | | |
