# Supplementary material for: A reasonable identification of the early recurrence time based on microvascular invasion for hepatocellular carcinoma after R0 resection: A multicenter retrospective study
Source: Cancer Med. 2023 Mar 6;12(9):10294–302. doi: 10.1002/cam4.5758 (PMC10225226; doi:10.1002/cam4.5758)
Supplement: Supplementary file 7 — Table S6 [file CAM4-12-10294-s004.docx]

| **Table S6.** Baseline characteristics of long-term recurrent HCC patients with MVI | | | |
| --- | --- | --- | --- |
| **Variables** | **LR+TACE**  **(n=52)** | **LR**  **(n=62)** | ***P*** |
| Age (year) |  |  | 0.960 |
| ≤ 55 | 34 (65.4%) | 37 (59.7%) |  |
| > 55 | 18 (23.6%) | 25 (40.3%) |  |
| Sex |  |  | 0.673 |
| Male | 44 (84.6%) | 51 (82.3%) |  |
| Female | 8 (15.4%) | 11 (17.7%) |  |
| WBC (*10^6^/L) |  |  | 0.398 |
| ≤ 4000 | 8 (15.4%) | 17 (27.4%) |  |
| > 4000 | 44 (84.6%) | 45 (72.6%) |  |
| RBC (*10^12^/L) |  |  | 0.425 |
| ≤ 4 | 8 (15.4%) | 13 (21.0%) |  |
| > 4 | 44 (84.6%) | 49 (79.0%) |  |
| PLT (*10^9^/L) |  |  | 0.935 |
| ≤ 100 | 11 (21.2%) | 20 (32.3%) |  |
| > 100 | 41 (18.9%) | 42 (67.7%) |  |
| PT (s) |  |  | 0.175 |
| ≤ 13 | 43 (82.7%) | 47 (75.8%) |  |
| > 13 | 9 (17.3%) | 15 (24.2%) |  |
| TBil (μmol/L) |  |  | 0.109 |
| ≤ 17.1 | 39 (75.0%) | 48 (77.4%) |  |
| > 17.1 | 13 (25.0%) | 14 (22.6%) |  |
| ALB (g/L) |  |  | 0.975 |
| ≤ 40 | 21 (40.4%) | 21 (33.9%) |  |
| > 40 | 31 (59.6%) | 41 (66.1%) |  |
| ALT (U/L) |  |  | 0.831 |
| ≤ 40 | 23 (44.2%) | 25 (40.3%) |  |
| > 40 | 29 (55.8%) | 37 (59.7%) |  |
| AFP (ng/mL) |  |  | 0.296 |
| ≤ 400 | 29 (55.8%) | 40 (64.5%) |  |
| > 400 | 23 (44.2%) | 22 (35.5%) |  |
| HBsAg |  |  | 0.097 |
| Positive | 49 (94.2%) | 51 (82.3%) |  |
| Negative | 3 (5.8%) | 11 (17.7%) |  |
| HBsAb |  |  | 0.231 |
| Positive | 3 (5.8%) | 10 (16.1%) |  |
| Negative | 49 (94.2%) | 52 (83.9%) |  |
| Child-Pugh class |  |  | 0.580 |
| A | 50 (96.2%) | 61 (98.4%) |  |
| B | 2 (3.8%) | 1 (1.6%) |  |
| Tumor diameter (cm) |  |  | 0.642 |
| ≤ 5 | 27 (51.9%) | 41 (66.1%) |  |
| > 5 | 25 (48.1%) | 21 (33.9%) |  |
| Note: HCC, hepatocellular carcinoma; MVI, microvascular invasion; WBC, white blood cell; RBC, red blood cell; PLT, platelet; PT, prothrombin time; TBil, total bilirubin; ALB, albumin; ALT, alanine aminotransferase; AFP, alpha-fetoprotein; HBsAg, hepatitis B surface antigen; HBsAb, hepatitis B surface antibody | | | |
|  | | | |
